# Human Umbilical Vein Endothelial Cells (HUVECs) Co-Culture with Osteogenic Cells: From Molecular Communication to Engineering Prevascularised Bone Grafts

**DOI:** 10.3390/jcm8101602

**Published:** 2019-10-03

**Authors:** Ievgeniia Kocherova, Artur Bryja, Paul Mozdziak, Ana Angelova Volponi, Marta Dyszkiewicz-Konwińska, Hanna Piotrowska-Kempisty, Paweł Antosik, Dorota Bukowska, Małgorzata Bruska, Dariusz Iżycki, Maciej Zabel, Michał Nowicki, Bartosz Kempisty

**Affiliations:** 1 Department of Anatomy, Poznan University of Medical Sciences, 60-781 Poznań, Poland; kocherova.evgenia@gmail.com (I.K.); abryja@ump.edu.pl (A.B.); m.dyszkiewicz@ump.edu.pl (M.D.-K.); mbruska@ump.edu.pl (M.B.); 2 Graduate Physiology Program, North Carolina State University, Campus Box 7608/321 Scott Hall, Raleigh, NC 27695, USA; pemozdzi@ncsu.edu; 3 Centre for Craniofacial and Regenerative Biology, Dental Institute, King’s College London, London WC2R 2LS, UK; ana.angelova@kcl.ac.uk; 4 Department of Biomaterials and Experimental Dentistry, Poznań University of Medical Sciences, 61-701 Poznań, Poland; 5 Department of Toxicology, Poznan University of Medical Sciences, 61-631 Poznań, Poland; hpiotrow@ump.edu.pl; 6 Veterinary Center, Nicolaus Copernicus University in Torun, 87-100 Toruń, Poland; pantosik@umk.pl (P.A.); dbukowska@umk.pl (D.B.); 7 Department of Cancer Immunology, Poznan University of Medical Sciences, Garbary 15 St., 61-866 Poznań, Poland; dmizy@ump.edu.pl; 8 Division of Histology and Embryology, Department of Human Morphology and Embryology, Wroclaw Medical University, 50-368 Wroclaw, Poland; mazab@ump.edu.pl; 9 Division of Anatomy and Histology, University of Zielona Góra, 65-046 Zielona Góra, Poland; 10 Department of Histology and Embryology, Poznan University of Medical Sciences, 60-781 Poznan, Poland; mnowicki@ump.edu.pl; 11 Department of Obstetrics and Gynecology, University Hospital and Masaryk University, 601 77 Brno, Czech Republic

**Keywords:** human umbilical vein endothelial cells, mesenchymal stem cells, osteoblasts, co-culture, and prevascularization

## Abstract

The repair of bone defects caused by trauma, infection or tumor resection is a major clinical orthopedic challenge. The application of bone grafts in orthopedic procedures is associated with a problem of inadequate vascularization in the initial phase after implantation. Meanwhile, the survival of cells within the implanted graft and its integration with the host tissue is strongly dependent on nutrient and gaseous exchange, as well as waste product removal, which are effectuated by blood microcirculation. In the bone tissue, the vasculature also delivers the calcium and phosphate indispensable for the mineralization process. The critical role of vascularization for bone healing and function, led the researchers to the idea of generating a capillary-like network within the bone graft in vitro, which could allow increasing the cell survival and graft integration with a host tissue. New strategies for engineering pre-vascularized bone grafts, that apply the co-culture of endothelial and bone-forming cells, have recently gained interest. However, engineering of metabolically active graft, containing two types of cells requires deep understanding of the underlying mechanisms of interaction between these cells. The present review focuses on the best-characterized endothelial cells—human umbilical vein endothelial cells (HUVECs)—attempting to estimate whether the co-culture approach, using these cells, could bring us closer to development and possible clinical application of prevascularized bone grafts.

## 1. Introduction

The repair of bone defects caused by trauma, infection or tumor resection, remains a major clinical orthopedic challenge. The application of autologous bone grafts, most commonly from the iliac crest, has been considered the gold standard. However, autologous bone grafts have some significant drawbacks, such as donor-site morbidity and graft size limitations. The procedure of autograft harvesting from the healthy bone increases the duration of surgery and can be associated with potential blood loss and risk of infection [1,2,3]. Additionally, autograft quality may be affected by patient’s age and metabolic disorders [4]. The inconsistent or low concentrations of endogenous mesenchymal stem cells (MSCs) can significantly decrease the efficacy of autograft transplantation. Therefore, bone tissue engineering approaches, which could help to overcome these problems, have recently gained interest. 

Advances in the field of regenerative medicine have stimulated the development of 3D tissue constructs comprised of the osteogenic precursors seeded on the osteoconductive carrier, also known as cellular bone matrices [5]. Although the engineered allografts may provide the advantages over the use of autologous bone grafts in orthopedic surgery, there is a problem of inadequate vascularization in the initial phase after implantation. Ingrowths of the host blood vessels within the 3D tissue constructs is often limited to several tenth of micrometers per day, and it may require several weeks to achieve the center of the implanted scaffold [6,7]. Moreover, newly formed vessels induced by inflammatory response are prone to the early regression [8]. Meanwhile, the survival of cells within the implanted graft and its integration with the host tissue is strongly dependent on nutrient and oxygen exchange, as well as waste product removal, which are provided by blood microcirculation. In the bone tissue, the vasculature also delivers the calcium and phosphate indispensable for the mineralization process [9]. Without pre-established vascular network, the transport of nutrients and oxygen occurs mainly by diffusion, which is limited to 100–200 µm from the host vasculature [10,11]. Successes in bioengineered tissue implantation are restricted to relatively thin or avascular structures, such as skin or cartilage because of the limited distance of oxygen diffusion. [10]. 

By contrast, bone is highly vascularized tissue, where angiogenesis precedes and is a pre-requisite for osteogenesis without regard to the type of ossification. In the process of endochondral ossification, forming the most bones of the skeleton, the hypertrophic chondrocytes release angiogenic growth factors that induce the blood vessels invasion within the cartilage. The new vasculature contributes to replacement of the cartilaginous template by bony callus. Endothelial cells constitute the inner lining of blood vessels and secrete the growth factors, controlling the recruitment of osteoclasts, osteoblasts and bone-forming cells [8,12]. Intramembranous ossification underlies the development of flat bones and clavicle, and also the formation of tissue-engineered bone grafts. During intermembranous ossification, bone tissue forms directly from osteoprogenitors condensations, without a cartilage intermediary. The endothelial cells incorporated into these condensations form vascular network serving as a “template” for bone mineral deposition [13,14,15]. Moreover, functional co-dependency between the osteogenesis and vessel formation occurs during not only the skeletal development, but also continuous bone remodeling and healing. 

The critical role of vascularization for bone functioning led the researchers to the idea of generating a capillary-like network within the bone graft in vitro, which could allow increasing the cell survival and graft integration with a host tissue. In vivo the formation of blood vessels is based on the two distinct processes—vasculogenesis and angiogenesis. Vasculogenesis refers to de novo assembly of endothelial progenitor cells (EPCs), their further differentiation to endothelial cells, proliferation and creation of the first primitive capillaries. Angiogenesis instead describes the formation of new capillaries from pre-existing blood vessels, which includes the migration of endothelial cells from the “mother” vessel [10,16]. Endothelial cells therefore represent an important participant in the both types of vessel formation processes. 

In the light of the above, a new strategy for engineering prevascularized bone grafts has been developed, that encompasses the co-culture of endothelial and bone-forming cells. Engineering of metabolically active graft, containing two types of cells is impossible without deep understanding of mechanisms of interaction between these cells. Here, we focus on the best-characterized endothelial cells—HUVECs—attempting to estimate whether the co-culture approach could bring us closer to development and effective clinical implementation of prevascularized bone grafts.

## 2. Cell Sources

### 2.1. Human Umbilical Vein Endothelial Cells (HUVECs)

Human umbilical vein endothelial cells (HUVECs) represent a widely used source of primary endothelial cells for in vitro studies of the vasculature and angiogenesis. HUVECs are relatively easy to isolate avoiding contamination by other cell types, and umbilical cord is readily available as discarded biological waste after the child’s birth. Furthermore, the umbilical vein lumen has an adequate capacity, allowing it to be easily cannulated and the endothelium can be detached by enzymatic digestion. Yuji Maruyama et al. was the first to report HUVECs primary culture in vitro obtained by perfusion of the umbilical vein with trypsin [17]. However, the scientific community interpreted Maruyama et al. [17] and some of the later attempts with skepticism, as the obtained cells were not definitively identified as endothelial cells [18]. Jaffe et al. [19], using similar enzymatic approach, successfully isolated the cells from the umbilical vein that could be identified as endothelial cells. It was shown that morphologically and immunologically identifiable human endothelial cells could be cultured in vitro for periods up to 5 months. Morphologic and immunologic criteria for endothelial cell identification can be [19]:

- Cell morphology (polygonal shape);
- The detection of Weibel-Palade bodies under electron microscopy;
- The presence of smooth muscle actomyosins;
- The expression of ABO blood group antigens.

In addition, cultured HUVECs contained antihemophilic factor antigen, now called von Willebrand factor (vWF) [20], which delineates endothelial cells from vascular smooth muscle cells and fibroblasts. It is important to note, that aforementioned Weibel-Palade bodies, occurring only in vascular endothelia, are the cytoplasmic components, which houses von Willebrand factor [21].

Several protocols for HUVECs isolation have been established [22,23,24,25]. Isolated HUVECs can be propagated using endothelial-specific media, supplements and optionally coatings for the culture vessels. Commercially available coatings, such as Matrigel (BD Biosciences) or Geltrex (Thermo Fisher Scientific), contain laminin, collagen IV, heparin sulfate proteoglycans, and entactins—compounds naturally occurring in basal lamina of blood vessels. As endothelial cells in vivo lie on a basal lamina, culture vessel coating can be a strategy for a cobblestone monolayer similar to vascular endothelium [23]. 

HUVECs have been reported to express many important endothelial markers and signaling molecules associated with regulation of vascular homeostasis [26,27]. In addition, HUVECs response to various physiological and pathophysiological stimuli, e.g., high glucose concentration, lipopolysaccharide (LPS) administration or shear stress [28,29,30]. HUVECs could also be successfully differentiated into 3D spheroids and co-cultured in 3D systems with other cell types, thereby enabling the establishment of advanced research models for better understanding of cell-to-cell communication and cell behavior in vivo. HUVECs have been used for vascularization of various engineered tissues, such as periosteum, bone tissue, and dental tissues, such as biomimetic tooth bud [31,32,33,34].

Furthermore, HUVECs are the most commonly used type of endothelial cells in the published researches on biomaterials [35]. Previous studies have shown promising results exploring the potential of in vivo application of HUVECs-containing grafts for biomaterials. For example, Mishra et al. reported that HUVECs develop functional vascular networks within poly(propylene fumarate) (PPF)/fibrin composite scaffolds, and these cultured, in vitro vessels, could further mature after implantation in vivo and connect to the host vasculature [36].

However, despite all their benefits, HUVECs do not necessarily represent a universal model of endothelial cells for every application (Table 1).

### 2.2. Other Sources of Endothelial Cells

The choice of vascular precursor cells is crucial for production of a functional vasculature within the in vitro engineered tissues. HUVECs are one of the most commonly employed mature ECs in vascularized bone engineering [37]. Another endothelial cell type that has gained attention are human dermal micro-vascular endothelial cells (HDMECs). Tissue harvest sites for HDMECs include small vessels within skin tissue from either adults or neonatal foreskins. Co-culturing of HDMECs and MSCs has been reported to ensure proliferation and differentiation of both cell types, osteogenesis stimulation and over-expression of angiogenesis-related genes in vitro [38]. Furthermore, an implantation of scaffolds pre-seeded with HDMECs and primary human osteoblasts led to perfused vessel formation and integration with host murine vasculature [39]. However, HDMECs as well as HUVECs represent fully differentiated cells, which includes their site-specific phenotype. Endothelial progenitor cells (EPCs) are characterized by a lack of phenotypic specificity. EPCs can be isolated in sufficient numbers from peripheral blood of adult patients and differentiated using ex vivo culture. EPCs were found to be more proliferative than HUVECs and have been recently used, also in combination with MSCs, to improve vessel formation in vivo and in tissue engineering applications [37]. However, there is still no consensus on phenotypic and functional definition of EPC [40]. Additionally, heterogeneity is also present in the vasculogenic potential of EPC subsets [41].

### 2.3. Mesenchymal Stem Cells 

Mesenchymal stem cells (MSCs) are the most promising type of cells, widely used in the studies on bone regeneration and investigation of tissue-engineered bone grafts. MSCs are gaining increasing attention during the last decade because of their unique biological properties. Ability to naturally differentiate into cells of mesodermal lineage in combination with auto- and paracrine action, that include the secretion of trophic and anti-inflammatory molecules, makes MSCs a promising tool for tissue repair and brought them at the forefront of regenerative medicine. Moreover, due to the low expression of major histocompatibility complex (MHC) class I molecules and lack of MHC class II and HLA-DR molecules, they appeared to be suitable for both autologous and allogeneic application [42]. MSCs meet the following minimal criteria proposed by The International Society for Cellular Therapy [43]:

- Adherence to plastic in standard culture conditions;
- Specific surface antigen expression, i.e., the presence of CD73, CD90, CD105 antigens, and lack of haematopoietic markers, inter alia CD34, CD45;
- Multilineage differentiation, which implies the ability of cells to differentiate into osteoblasts, chondroblasts and adipocytes with appropriate stimuli in vitro.

MSCs were initially isolated from bone marrow, but subsequent research revealed the presence of MSCs in a large number of adult organs and tissues, as well as body fluids [44]. In the extramedullary niches, MSCs are believed to serve as a reserve to replace damaged and senescent cells and are recognized for their potential clinical application in tissue repair. 

Bone marrow and adipose tissue are the most common stem cell sources currently used in regenerative medicine. However, both bone marrow (BM-MSCs) and adipose tissue-derived MSCs (ASCs) exhibit limitations, as their proliferation and differentiation abilities can be affected by donor’s age and underlying pathologies [45,46,47]. The regenerative potential of tissue resident MSCs has been reported to decline after 30 years of age [48], and their exhaustion is considered to be an important component of aging [49,50]. By contrast, Wharton’s jelly derived-mesenchymal stem cells (WJ-MSCs), seem to be devoid of these disadvantages, as the umbilical cord represents a young fetal tissue [51]. However, although MSCs isolated from different organs and tissues possess different regenerative potential associated with the environment of stem cell niche, they share the ability to differentiate into osteogenic cells.

Osteogenic cells differentiate and develop into osteoblasts which, in turn, are responsible for forming new bones.

Osteoblasts (OBs) have gained much interest in the field of bone tissue engineering as they secrete bone matrix proteins, including collagen type 1 alpha 1 (Col1α1), alkaline phosphatase (Alp), osteocalcin (OC). Their activity is known to be highest during embryonic skeletal formation and growth [52]. Despite some successful attempts to obtain microcapillary-like structures via the co-culture of endothelial cells and osteoblasts, the latter might not be good source of transplanted cells because they are not multipotent, being already differentiated from MSCs [53]. However, the studies on ECs-OBs co-culture may shed a light on the interaction of these two types of cells in vivo.

## 3. Cell Communication

**Figure 1:**
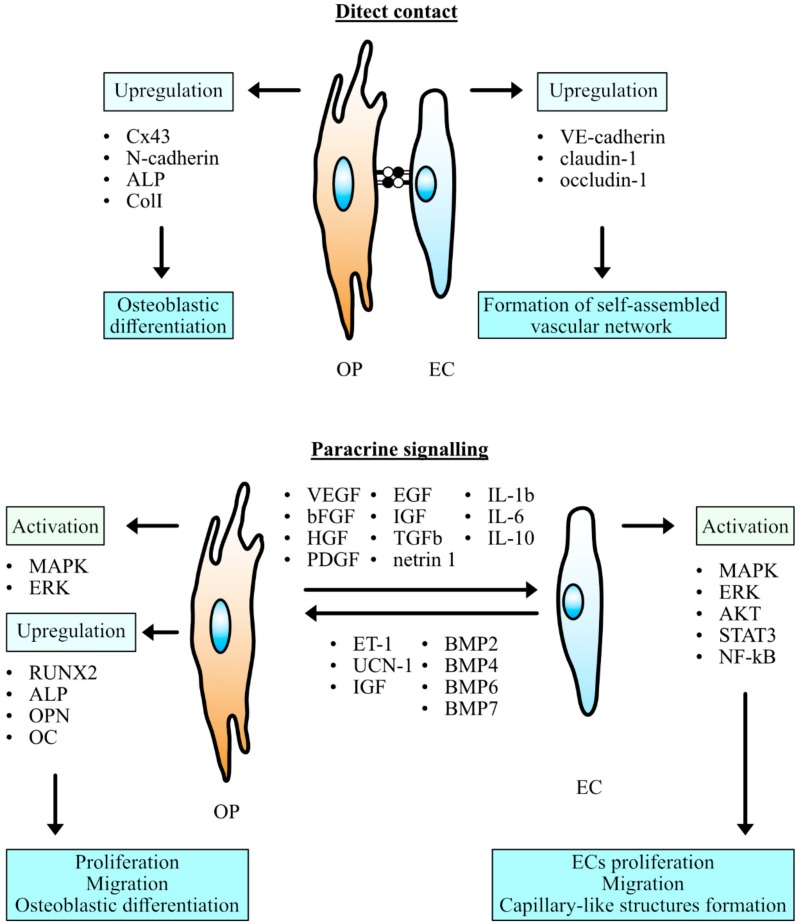
Communication between the osteogenic precursors (OP) and endothelial cells (EC). MAPK mitogen-activated protein kinases, ERK extracellular-signal-regulated kinase, AKT protein kinase B, STAT3 signal transducer and activator of transcription 3, NF-kB nuclear factor kappa-light-chain-enhancer of activated B cells, RUNX2 Runt-related transcription factor 2, ALP alkaline phosphatase, OPN osteopontin, OC osteocalcin, VEGF vascular endothelial growth factor, bFGF basic fibroblast growth factor, HGF hepatocyte growth factor, PDGF platelet-derived growth factor, IGF insulin-like growth factor, TGFb transforming growth factor beta, IL interleukin, ET-1 endothelin-1, UCN-1 urocortin 1, BMP bone morphogenetic protein.

In recent years, an increasing number of research studies have examined an interaction between endothelial cells and osteoprogenitors which are always located in close proximity to each other at sites of bone formation indicating an important role of their proper communication in bone healing and remodeling [54]. Several mechanisms of cell-to-cell communication were supposed to underlie reciprocal regulation and functional relationship between the endothelial and osteogenic cells. Intercellular communication is supported by direct contact between adjacent cells, through membrane molecules and gap junctions, along with paracrine signaling, which requires diffusible factors spreading through the extracellular matrix (Figure 1) [6]. Endothelial cell properties and the manner of their crosstalk with co-cultured cell lines may be dependent on their source [8]. Therefore, the present review focuses mainly on the reports concerning HUVECs implementation in the context of co-culture with osteoprogenitor cells, as HUVECs are one of the most available and well-studied types of vascular endothelial cells.

### 3.1. Dynamics of Adherens Junctions in Co-Cultured Cells

Direct interaction between the membranes of adjacent cells plays a crucial role in endothelial adhesion and barrier function, which is important for vessel formation and maintaining tissue homeostasis. In bone, this type of direct intercellular communication controls osteoblastogenesis and osteoblast differentiation [55,56]. Adherens junctions couple individual cells into various arrangements using homophilic Ca^2+^-dependent interactions of transmembrane molecules called cadherins. Some of them have drawn special attention in the studies concerning co-culture of endothelial and osteogenic cells.

#### 3.1.1. VE-Cadherin

Vascular endothelial (VE)-cadherin is a strictly endothelial-specific adhesion molecule. The extracellular domain of VE-cadherin possesses multiple binding sites for calcium and is responsible for binding with cadherin expressed on the surface of neighboring cells. The cytoplasmic domain binds β-catenin and plakoglobin, and both molecules in turn can bind α-catenin, which through the association with F-actin contributes to regulation of cell shape and motility [57,58]. However, there are the studies that have questioned this model of junction’s linkage to the actin cytoskeleton by suggesting more complex cadherin-mediated regulation of actin assembly [59,60]. Besides the action on cytoskeleton, VE-cadherin/β-catenin signaling controls endothelial cell survival, and is indispensable for vessel formation, since the inhibition or depletion of VE-cadherin has been reported to increase endothelial apoptosis and permeability, vessel collapse and major defects in vascular remodeling [61,62]. 

**Figure 2:**
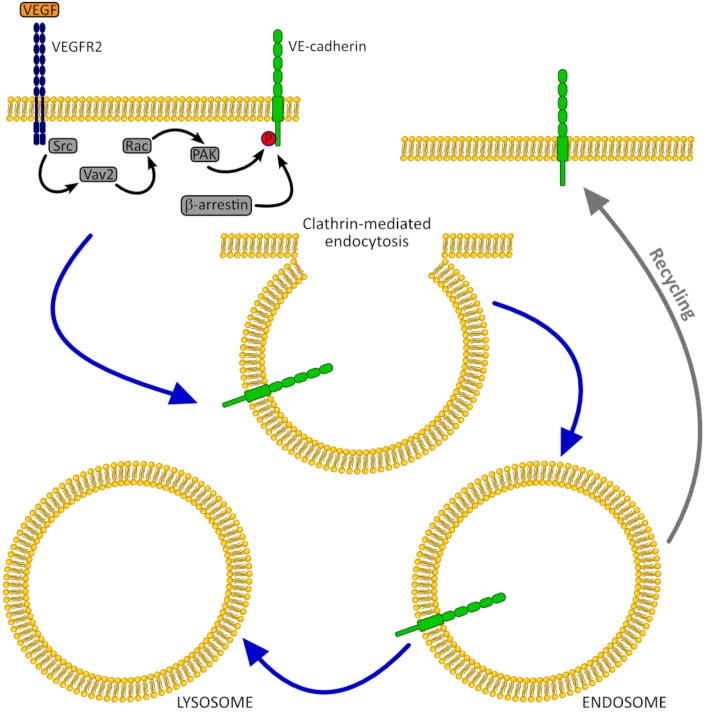
VE-cadherin internalization caused by stimulation with vascular endothelial growth factor (VEGF). VEGF binding to VEGFR-2 results in dimerization of this receptor, followed by Src-dependent phosphorylation of the guanine nucleotide exchange factor Vav2, subsequent activation of small GTPase Rac, and its downstream effector, the serine/threonine protein kinase PAK. This leads to serine phosphorylation of VE-cadherin cytoplasmic tail, followed by β-arrestin2 recruitment and VE-cadherin internalization into clathrin-coated early endosomes [64,67,68].

Several studies have shown that co-culture with MSCs may influence the expression of VE-cadherin and its membrane localization in HUVECs. Thus, Chen et al. in the study on HUVECs co-cultured with MSCs from different origins observed significant up-regulation and peak of VE-cadherin gene expression at 4 weeks [53]. However, after 6 weeks of culture VE-cadherin expression reverted to the basal level. Li et al. reported the increase of VE-cadherin transcript and protein levels in HUVECs co-cultured with bone marrow-derived MSCs (BM-MSCs) after 24 h, when compared with those in HUVECs monoculture. Interestingly, despite VE-cadherin expression remained unchanged at earlier time points, immunofluorescence staining showed low VE-cadherin engagement in cell junctions after 6 h and 14 h of HUVECs/BM-MSCs co-culture, whereas in HUVECs monoculture VE-cadherin was detected at junctions all the time. This temporary loss of VE-cadherin from cell junction might be due to VEGF stimulation, which was reported to be highly expressed in co-cultured BM-MSC [63]. Indeed, in recent study on HUVECs, exposition to VEGF has been shown to promote VE-cadherin phosphorylation, causing its endocytosis and junction weakening [64]. However, this process is reversible, and internalized VE-cadherin is able to relocalize to the cell surface and participate in adherens junctions (Figure 2). 

The dynamic localization of VE-cadherin in HUVECs membrane was also described in the study on human umbilical mesenchymal stem cells (WJ-MSCs) transmigration across HUVECs monolayer [65]. WJ-MSCs promote discreet VE-cadherin perturbation preceding paracellular transmigration followed by up-regulation of both VE-cadherin protein expressions leading to adherens junctions at the subendothelial position.

Interestingly, while VEGF release, at least in part, may represent a mechanism where MSCs promote temporary loss of VE-cadherin from HUVECs junctions, co-culture with MSCs can prevent disorganization of adherens junctions induced by exposure to exogenous VEGF. Thus, Pati et al. reported that exogenous VEGF enhanced endothelial permeability only in HUVECs monoculture, whereas co-culture with MSCs had permeability-stabilizing effect and increased VE-cadherin membrane localization [66]. Furthermore, the research group found that conditioned medium from HUVECs/MSCs co-culture, but not from HUVECs or MSCs cultured alone, can enhance VE-cadherin levels and reduce paracellular permeability. Based on these observations, it can be assumed that MSCs-HUVECs interaction in co-culture may lead to the production of some soluble factors that have barrier protective properties. 

In general, while up-regulation of VE-cadherin in HUVECs/MSCs co-culture can ensure the formation of self-assembled network, the temporary junctions disassembly, reported in several studies, is indispensable for cell migration and vascular remodeling.

#### 3.1.2. N-Cadherin

Whereas VE-cadherin is known to mediate adhesion only between endothelial cells, there is another member of cadherin family, called neural (N)-cadherin, which has been reported to be widely expressed in multiple tissues and participate in heterotypic contacts between different cell types [56,57]. The cytoplasmic domains of VE-cadherin and N-cadherin are highly homologous and share some intracellular partners such as b-catenin or p120-catenin [57]. However, in contrast to VE-cadherin, which is located at adherens junctions, N-cadherin is distributed over the entire cell membrane [56]. 

N-cadherin has been shown to be overexpressed in co-cultures of BM-MSCs with HUVECs, which resulted in improved cell-cell adhesion in comparison to monocultures [69]. In addition, co-cultured HBMSCs have been characterized by enhanced translocation of N-cadherin from the cytoplasm to the cell membrane. 

Moreover, up-regulation of N-cadherin expression promotes the production of both ALP and type I collagen in co-cultured BM-MSCs. The mechanisms enhancing osteoblastic markers through N-cadherin expression remain unclear, although the possible link between N-cadherin and connexin 43 (Cx43), has been suggested to underline this effect [69,70]. Another study conducted on preosteoblasts monoculture has revealed that correlation between N-cadherin levels and expression of osteoblast differentiation markers might be supported by PI3K signaling [71]. 

Recently, it has been hypothesized that N-cadherin exerts different effects on osteogenic lineage cells depending on their differentiation stage [72]. Moreover, while cadherin interactions develop progressively and promote osteoblast differentiation throughout the early stages of osteogenesis, terminally differentiated bone forming cells were shown to lose their cell-cell adhesion [70,73]. To date, there is no data available on N-cadherin dynamics in pre- and mature osteoblasts co-cultured with HUVECs. Thus, the question whether the effects on N-cadherin expression induced by co-culture with HUVECs are limited to MSCs, remains to be addressed.

### 3.2. β-Catenin Signaling

**Figure 3:**
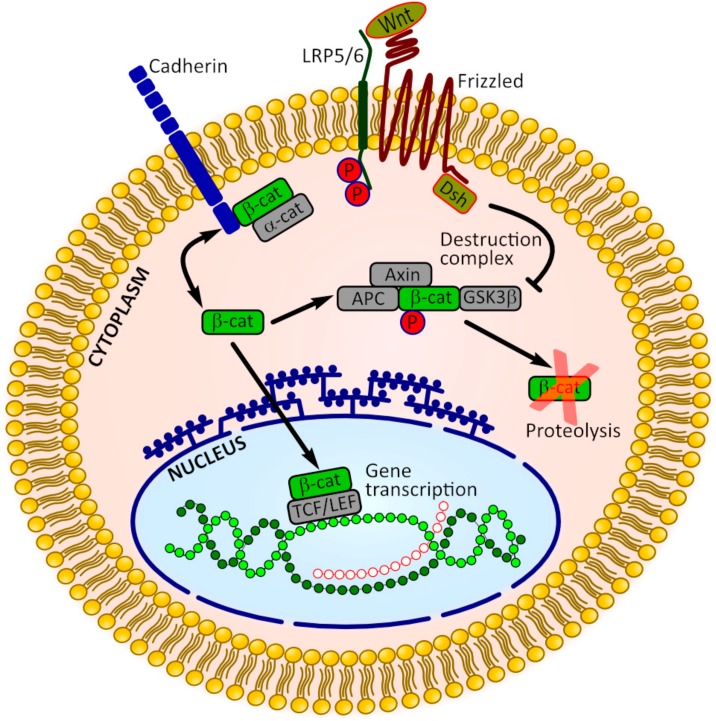
The interactions between cadherin-mediated adhesion and Wnt/β-catenin signaling. Cadherins bind β-catenin molecules, sequestrating them at the cell membrane. Adherens junctions weakening and disruption of cadherin/β-catenin binding results in the the release of β-catenin into the cytoplasm, making more β-catenin available in transcriptionally active pools. In the absence of Wnt signaling β-catenin levels are kept in check by the so-called destruction complex comprising, among others, adenomatous polyposis coli (APC), axin and glycogen synthase kinase 3β (GSK3β). Wnt binding to the receptors LRP5/6 and Frizzled results in the inhibition of GSK3β activity and disruption of degradation complex, followed by β-catenin accumulation and translocation into the nucleus, where it binds T-cell factor/lymphoid enhancer factor (TCF/LEF) family of transcription factors and to induce the expression of target genes [74,75].

Besides the key role in stabilization of adherens junctions, β-catenin acts as a transcriptional co-factor downstream of the canonical Wnt signaling involved in regulation of cell proliferation and differentiation. Thus, β-catenin represents a convergence point where cadherins can interact with Wnt signaling (Figure 3). For example, b-catenin that is associated with cadherins anchored in the cell membrane contributes to the elevation of b-catenin in the cytoplasm when cadherin internalization occurs. Destabilization of the cadherin-β-catenin binding may increase the transcriptionally active pool of β-catenin, lowering the threshold for Wnt signaling. In contrast, stabilization of cadherin function is supposed to diminish the cytoplasmic pool of b-catenin and attenuate canonical Wnt signaling. Similarly, the excessive accumulation of cytoplasmic b-catenin may promote its saturated binding to cadherin molecules in the cell membrane, leading to the strengthening of adherens junctions [55,74,75]. 

The experiments conducted on HUVECs co-cultured with osteoprogenitor cells have also demonstrated the interaction between cadherin-β-catenin binding and Wnt signaling. The study of Menge et al. revealed that co-culture with MSCs exerted profound stabilizing effects on HUVECs, which included enhanced localization of VE-cadherin and β-catenin at the cell membrane [76]. The co-culture with transfected Wnt3a-expressing MSCs or the addition of the exogenous Wnt3a abrogated these effects and diminished the pool of membrane-bound β-catenin in HUVECs. These observations are consistent with those of Pati et al., who reported that the protein levels of active, dephosphorylated b-catenin were decreased, whereas VE-cadherin levels were increased in HUVECs after co-culture with MSCs [66]. Additionally, HUVECs co-cultured with MSCs showed a remarkable reduction in T-cell factor/lymphoid enhancer factor (TCF/LEF) reporter transcription compared with those in HUVECs monoculture. The inverse correlation between adherens junction’s formation and Wnt signaling has also been shown in co-cultured osteogenic cells. Thus, the increase of N-cadherin mRNA level has been accompanied by the decrease of TCF-1 gene expression in BM-MSCs co-cultured with HUVECs for 48h [69].

### 3.3. Communication Through the Gap Junctions

Cadherin-based adhesion between the adjacent cells is critical for the formation of gap junctions [75,77] representing a direct cytoplasmic connection through the specialized hemichannels embedded in the membranes of apposed cells. Each hemichannel, or connexon, is composed of six connexin (Cx) monomers arranged side-by-side. Connexin isotypes involved in formation of the resulting gap junction channel determine its molecular size and permeability.

There are four connexins found to be expressed in the vascular endothelium: C×32, Cx37, Cx40 and the most prevalent Cx43. Recent data has suggested that Cx32 and Cx43 in HUVECs contribute more to the functional phenotype of endothelial cells than do Cx37 and Cx40 [78]. Apart from forming gap junction, Cx43 protein also participates in the modification of cell cycle and regulation of transcription. Moreover, Cx43 has been suggested to modulate F-actin cytoskeletal architecture and cellular migration in wounded endothelium through the coupling with the tight junction protein zonula occludens (ZO)-1 [77,79]. Reduced expression of Cx43 gap junctions may be a potential indicator of endothelial dysfunction [80].

In osteoprogenitor cells, gap junctions are considered to influence the cell fate. Inhibition of gap junctional communication among human osteoblasts has been shown to result in not only the loss of osteogenic phenotype but also the trans-differentiation of cells toward the adipogenic lineage, despite of osteoinductive culture conditions [81]. In bone, as well as in the endothelium, the predominant gap junction protein is Cx43. Additionally, the connexons formed by Cx45 are also present in osteogenic cell membrane, however, to a lesser extent. 

Among the connexins expressed by both HUVECs and cells of osteogenic lineage, Cx43 is also considered to be most closely associated with the process of osteogenesis and bone formation. Cx43 gap junctions are permeable to molecules of up to 1.2 kDa, which allows the passage of ions, metabolites and second messengers such as calcium, cyclic nucleotides, and inositol derivatives [75]. It has been well documented that communication through Cx43 gap junctions allows osteoprogenitor cells to express high levels of both early osteoblastic differentiation markers, such as alkaline phosphatase (ALP), and markers of late osteoblastic differentiation, such as osteocalcin (OC), and bone sialoprotein (BSP) [6,54,82,83]. 

Villars et al. demonstrated for the first time that HUVECs and BM-MSCs coupled through the gap junctions composed of the Cx43 [82]. Immunofluorescence staining for Cx43 and vWF revealed a specific labeling of Cx43 between a vWF-positive cell and a vWF-negative cell in some areas of the co-culture, which suggested heterotypical gap junctional connection between HUVECs and HBMSCs. Furthermore, a functional coupling between these types of cells was confirmed by transfer of the intercellular fluorescent dye Lucifer yellow, which is commonly used for tracing connections between cells. 

Intercellular passage of signaling molecules through the junctional channel has been hypothesized to support osteogenic differentiation in co-cultures of endothelial and osteoprogenitor cells. There are several reports showing that the expression of osteoblast marker ALP was higher in co-cultures, when compared to those in HUVECs or osteoprogenitor cells cultured alone [83,84,85]. The inhibition of Cx43 was shown to abolish this effect, suggesting that Cx43 gap junctions were necessary for the stimulation of ALP activity in this co-culture model. By contrast, the latter study of Guillotin et al. revealed the decrease of the Cx43 protein abundance with simultaneous up-regulation of the ALP expression in co-culture of HUVECs with osteoprogenitor cells [54]. Given the contradiction of these findings with the widely accepted assumption about Cx43 role in expression of osteoblast markers, the authors hypothesized, *inter alia*, that down-regulation of Cx43 could be balanced by an increase in gap junctional activity [54].

### 3.4. Paracrine Action

Since one of the earlier studies [86] on HUVECs co-cultured with osteoblasts suggested the model of bilateral communication through the diffusible factors, there has been growing body of research supporting the existence of mutual relationships between these types of cells. Growth factors produced by endothelial cells include endothelin-1 (ET1), insulin-like growth factor (IGF), bone morphogenetic protein-2 (BMP2), which can affect osteogenic cells behavior through the interaction with the appropriate membrane receptors [6]. In one of the recent studies, Steiner et al. have observed an increase in osteoblasts and MSCs proliferation in the insert-based indirect co-culture with HUVECs under low-serum conditions suggesting that insulin-like growth factor-1 (IGF-1) secreted by endothelial cells in culture medium could underlie this effect [87]. HUVECs have also been shown to express and secrete peptides belonging to corticotropin-releasing hormone (CRH) family, such as urocortin 1 (Ucn1) and other factors known to possess antioxidant and immunomodulatory properties [88]. 

Mesenchymal stem cells and osteoblasts also secrete a variety of soluble factors, including classic angiogenic factors, such as vascular endothelial growth factor (VEGF), basic fibroblast growth factor (bFGF), transforming growth factor beta (TGFb) [89]. Moreover, accumulating evidence indicates that the healing effects of MSCs in vivo are mainly related to their unique paracrine properties rather than ability to engraft and differentiate at injured sites [90,91,92,93,94,95]. 

The application of conditioned medium is one of the widely used approaches to investigate the paracrine effects of different cell types. Chen et al. have shown that BM-MSCs-conditioned medium significantly enhanced HUVECs migration and proliferation when compared to fibroblast-conditioned medium [96]. Furthermore, the proliferation-promoting effect of conditioned medium arising from either BM-MSCs or osteoblasts may be associated with the angiogenic action of VEGF synthesized by osteoprogenitor cells [82,86]. Prasadam et al. reported that VEGF secreted from the osteocyte cell line improved proliferation and migration of HUVECs and stimulated angiogenesis through the activating VEGFR2–MAPK–ERK-signaling pathway in endothelial cells. Inhibiting VEGF or MAPK–ERK pathways abolished these osteocyte-mediated effects in HUVECs [97,98]. 

Taking into account that tissue-engineered grafts appear to be exposed to pathological conditions after the transplantation to the site of injury, Zhang and colleagues investigated the effect of hypoxic microenvironment on cultured cells relevant to that characteristic for avascular bone necrosis [98]. Conditioned medium derived from MSCs under hypoxic conditions improved the survival rate, migration and angiogenesis in HUVECs, whereas apoptosis rate was inhibited [98]. The analysis of conditioned medium revealed the up-regulation of SDF-1α, VEGF and IL-6 levels, when compared to those in medium derived from HUVECs and MSCs cultured under the normal conditions. Moreover, a recent study conducted by Prieto et al. demonstrated that WJ-MSCs produce not only classical angiogenesis-related factors, such as VEGF, but also netrin-1 serving as a pro-angiogenic regulator of endothelial cells [99]. Thus, netrin-1, present in WJ-MSC-conditioned medium at low amounts, similar to those established in the umbilical cord blood, has been shown to promote tubule formation by HUVECs. Pharmacological blockage of netrin-1 in WJ-MSCs has been found to result in diminished angiogenesis in HUVECs. Moreover, using the exogenous netrin-1 the researchers confirmed that only physiological concentrations were beneficial for initiation of angiogenic process, as higher concentrations tends to promote endothelial cell migration to a lesser extent.

MSCs have also been reported to produce extracellular vesicles (EVs) rich in various bioactive molecules able to exert anti-inflammatory, antioxidant and proliferative effects. Therefore, it is possible that development of novel cell-free, secretome-based therapies may bypass the difficulties associated with stem cell therapy [100]. Apart from different growth factors, EVs have been shown to comprise functional small RNAs. EVs can move to the distant sites, where their content may be utilized by other cells, supporting wound healing and tissue regeneration *in vivo*. In turn, under in vitro conditions, EVs accumulate in culture medium and can be subsequently isolated using ultracentrifugation [101]. It has been suggested that MSCs-derived exosomes undergo internalization by HUVECs, which promotes tube-like structures formation by endothelial cells in vitro [102,103]. Pro-angiogenic microRNAs in the exosomes transferred to HUVECs may lead to the formation of endothelial tubes [103]. Furthermore, MSCs-derived exosomes were found to activate several important signaling cascades in target cells, including ERK, AKT and STAT3 [102]. 

Application of conditioned medium or isolated extracellular vesicles does not allow the full investigation of the paracrine effects, such as the secretion of soluble factors that may be dependent on the direct contact between the co-cultured cells. For instance, the study of Li et al. revealed the induction of IL1b or IL6 synthesis in HMSCs caused by direct contact with HUVECs [104]. These two interleukins were shown to activate NF-kB in co-cultured HUVECs, which was associated with the promotion of angiogenic processes, such as formation of cell aggregation structures. Neither indirect co-culture with the use of semipermeable insert membranes nor conditioned medium could achieve the significant induction of these interleukins level. 

### 3.5. Role of Extracellular Matrix in Cell-Cell Communication

Extracellular matrix (ECM) has a significant impact on intercellular communication through paracrine signaling, because of its ability for entrapping and storage of bioactive molecules secreted by the cells. Heparan sulfate proteoglycans occurring in ECM are considered to bind a wide variety of proteins, such as diffused growth factors, chemokines and enzymes [105]. In turn, cleavage of ECM proteins by matrix metalloproteinases (MMPs) release ECM-bound effectors molecules, allowing them to exert their biological role [106]. ECM, apart from providing structural support, contributes to the regulation of cell phenotype, proliferation, migration, and physiology [107]. Due to its beneficial properties and ability to guide cell behavior, ECM can also be used as a biological scaffold [6,108]. 

The components of ECM have been suggested to support interactive relationships between endothelial and osteoprogenitor cells. Villars et al. reported that ECM derived from HUVECs enhanced BM-MSCs proliferation rate in comparison to HBMSCs control cultured on the plastic or on their own ECM. However, HUVECs-derived ECM appeared to have no effect on ALP activity in HBMSCs, when compared to HBMSCs cultured alone on plastic, and to HBMSCs co-cultured with HUVECs with direct contact [109]. By contrast, Kang et al. observed significantly increased ALP activity and up-regulated expression of osteogenesis-related genes such as RUNX2, ALP, osteopontin (OPN) and osteocalcin (OC) in HBMSCs cultured on ECM-containing β-TCP scaffold in comparison to control group seeded onto β-TCP scaffold alone [110]. These interactive effects may be mediated by MAPK/ERK signaling pathway, since the inhibition of ERK resulted in down-regulation of aforementioned osteogenesis-related markers. 

Osteogenic ECM collected at the phase of maximal deposition and glycosylation of collagen I is able to induce the formation of vessels-like structures in HUVECs culture demonstrating the effect of ECM derived from mature osteoblasts [111]. The activation of p-38/MAPK signaling pathway was shown to underlie this effect. 

Interestingly, direct co-cultivation with osteogenic cells has been reported to improve ECM synthesis by HUVECs. Thus, a microarray analysis performed by Simunovic et al. revealed a markedly increased transcription of ECM-related genes in HUVECs following co-cultivation with osteoblasts [112]. In a more recent study, ECM produced by HUVECs increased the expression of alkaline phosphatase (ALP) in osteoblasts [113]. Further investigations are needed to delineate the exact components of ECM and signaling pathways that are involved in this interaction.

## 4. Co-Culturing Systems and Optimization of Conditions

Different co-culturing systems, using HUVECs have been studied and developed through the years, mainly focusing on two widely accepted approaches as the direct and indirect co-culture systems. The direct co-culture approach is based on simultaneous seeding of two adherent cell types on the culture plastics, set up as 2D monolayer or seeded as spheroids on special low adherent dishes to establish 3D system. The indirect co-culture approach is based on using “*inter alia*”, seeding the cells on separate levels using semi-permeable membranes that enable exchange of ECM derivates and signaling in some systems. Regardless of the approach chosen, there are always challenges in optimizing the condition regarding the cell seeding density and ratio, as well as conditioning of the growth medium, balancing and providing synergistic growth of the co-cultured cells.

Shah et al. suggested that co-cultures of HUVECs and OBs in ratios of 5:1 positively influenced vasculogenesis, while 1:5 ration of HUVECs:OB influenced higher rate of mineralization [114]. This study was using monolayer co-culturing system. In turn, De Moor et al. reported that HUVECs could form viable and stable spheroids when combined with fibroblasts or ASCs in a 1:9 cell ratio [115]. Spheroids with a greater HUVECs proportion were characterized by poor aggregation and morphology. In line with this result is the previous study by Ma et al. who suggested that a higher HUVECs proportion (>50%) in co-culture with MSC, prevented the creation of spheroids [116]. This study also suggested that a HUVECs:MSCs co-culture ratio of 1:1 is the best combination to obtain both osteogenic and angiogenic differentiation. These results are consistent with that obtained in the other studies, where 1:1 ratio provided robust and stable vascular networks while enabling bone-like tissue formation [15,117].

The composition of growth medium also influences phenotype and behavior of the cells grown in the co-culturing systems. One of the earlier studies on HUVECs revealed that endothelial cells cultured in supplemented Endothelial Cell Growth Media (EGM) expressed Vascular Endothelial Factor (VEGF) receptors constitutively, whereas cells cultured in alpha MEM without EGM demonstrated a time-dependent decrease in the level of VEGF receptor gene expression [86]. The more recent study of Correia et al. has demonstrated that HUVECs did not survive when the osteogenic medium was applied at the very beginning of co-culturing with MSCs [15]. It has been found that, for co-localized development of capillary-like and bone-like tissues, HUVECs-MSCs co-culture should first be supplied with EGM, followed by the application of cocktail medium composed of EGM and osteogenic medium at 1:1 ratio. Additionally, supplementation of cocktail medium with bone morphogenetic protein-2 (BMP-2, 10 ng/mL) was shown to significantly decrease the amount of cell debris, suggesting that BMP-2 may support the cell viability. 

In a co-culture of endothelial cells (ECs) and MSCs, angiogenic supplements stimulate the formation of primitive vascular networks by the ECs. These, in turn, recruit MSCs into the creation of pericyte-like coverage of endothelial tubes that enables the immature vessels to remain stable during the osteogenic stimulation [15,118]. It has been shown that the capillary-like structures formed by HUVECs were stable in the presence of MSCs due to their pericyte-like role in this system [15]. Thus, sequential induction of vascular formation and osteoprogenitor cells maturation seems to be beneficial to facilitate the development of both structures within single graft for bone tissue engineering applications.

## 5. In Vivo Studies

The potential of combining osteogenic cells with endothelial cells to increase the vascularization of bone grafts has been examined in several in vivo studies. Koike at al. successfully created long-lasting blood vessels in vivo by co-implanting HUVECs and 10T1/2 mesenchymal precursor cells in fibronectin–type I collagen gel [119]. The vascular network remained stable and functional for one year after the graft implantation in mice. The authors observed a rapid increase in the number of perfused vessels in the first two weeks. By contrast, the grafts prepared from HUVECs alone showed minimal perfusion and disappeared after 60 days. Immunohistochemistry revealed that the 10T1/2 cells differentiated into mural cells. In turn, some mural cells of the newly-formed vessels derived from the host. The engineered vessels, which responded appropriately to vasoactive stimuli. The similar results were obtained by Au et al. [120]. Additionally, authors reported that hMSCs did not differentiate to endothelial cells in vitro, and hMSCs by themselves could not form conduit for blood flow in vivo. The recent study of Ma and colleagues demonstrated that co-cultures using either human bone marrow- or human adipose tissue-derived MSCs with HUVECs have equal angiogenic capacity both in vitro and in vivo, and that vessels from donor origin can anastomose with the host murine vasculature within seven days after implantation [121]. Whereas the aforementioned studies applied the collagen gels for 3D grafts creation, there is a study examining another type of 3D construct composed of homotypic or heterotypic cell sheets [122]. The authors used an ectopic implantation model in rats. The study revealed that double osteogenic cell sheet constructs, with HUVECs in between, led to a faster and more robust formation of bone tissue, when compared with constructs without HUVECs. It was also shown that the implanted constructs induce bone formation through the recruitment of host’s osteogenic cells. However, the described in vivo experiment lasted only 7 days, and there is a question regarding the stability of this type of constructs during longer period. 

## 6. Issues of Concern and Future Perspectives

Despite the fact that primary isolated HUVECs are the best-characterized type of endothelial cells used in research, there is still a lack of information how the usage of these cells will address the phenotypic and functional heterogeneity of endothelium from different vascular origins [123]. HUVECs represent fully differentiated cells, which cannot further develop into organ-specific endothelium [124]. Thus, the quality of bioengineered vessels formed by HUVECs may be compromised due to lack of organ specificity. For example, HUVECs are considered to be inappropriate cell type for nervous tissue vascularization, as they are not capable to form the tightly sealed endothelium, and establish an effective blood-brain barrier, which is specific to the central nervous system [125]. The barriers formed in vitro by HUVECs have been characterized by higher permeability than that consisted of other types of endothelial cells [126,127]. However, the bone sinusoidal wall is known to be perforated by inter- and intra-endothelial pores, which support the passage of mature hematopoietic cells towards the blood circulation, as well as extravazation of intravascular, relatively large molecules [128]. Thus, “leaky” HUVECs-derived endothelium does not pose as a problem in the case of bone tissue engineering. 

Among all of the available endothelial cell types, HUVECs uniqueness lies in the fact that they originate from the vessel existing only at a certain stage of ontogenesis. The umbilical vein supplies the fetus with blood, rich in oxygen and nutrients, providing the opportunity for the endothelium to be exposed to higher oxygen concentrations compared with veins in other organs. Therefore, HUVECs may not represent a relevant model of endothelium, as oxygen concentration influences cellular functions, such as proliferation, chemotaxis, and tubulogenesis [35,129]. Although the bone marrow is highly vascularized tissue, its blood perfusion is heterogeneous and some regions are poorly perfused and hypoxic, compared with others. Spencer et al. revealed that within endostium, where most of the smaller vessels are located, the partial oxygen tension (PO_2_) levels were 21.9 mmHg (2.9%) in the vessels and 13.5 mmHg (1.8%) outside the vessels. The lowest PO_2_ (~9.9 mmHg, or 1.3%) was reported in deeper peri-sinusoidal regions [130]. By contrast, fetal umbilical vein PO_2_ has been reported to range from 22 to 53 mmHg [131,132]. 

However, endothelial cells in vitro differ from their in vivo counterparts, and the difference appears to increase as the cells continue to divide [129]. Therefore, culture conditions, can significantly affect the cell phenotype. Cells cultured in the laboratory are exposed to room air oxygen concentration of 21%, which corresponds to a PO_2_ of approximately 150 mmHg at sea level [133]. Maintaining the cultured cells at PO_2_ is considered to be normoxic, despite the obvious difference between the air and tissue PO2 levels. Thus, regardless the oxygen tension in the vascular of origin, endothelial cells may change their properties in vitro under the influence of room air oxygen. However, this issue needs to be further studied in the light of these considerations. 

The next intriguing issue concerns the gender differences described between HUVECs obtained from female and male umbilical cords. These involve, in particular cell proliferation and migration, nitric oxide synthase expression, as well as the level of autophagy [134]. However, none of the studies reviewed took into account this phenomenon. 

In addition, although the studies revealed that HUVECs-derived vessels after implantation in vivo are capable of connecting with host circulatory system, there is a question of endothelial activation and graft reseeding with the host endothelium [135]. 

Finally, despite the large number of studies, a comprehensive picture of the processes accompanying the interaction between endothelial and osteogenic cell types does not yet exist. Many of the mechanisms supporting this interaction remain not fully elucidated due to the complexity of in vivo environment and limitations of in vitro studies. Moreover, the simultaneous formation of capillary-like network and mineralized deposits in vitro within a single culture environment is a challenge, even as the linkage of vascular development and osteogenesis in vivo seems to be well understood. 

The research findings clearly indicate that the strategy of engineering bone grafts comprising the cells of osteogenic and angiogenic lineages has great potential for orthopedic treatment. Pre-vascularized engineered graft implantation can overcome the limitations of using autografts for bone repair. However, lack of organ-specificity and the differentiation stage of HUVECs should be considered. The future research work should focus on providing a better understanding of the interactions between HUVECs and osteogenic precursors, the underlying mechanisms that influence these interactions as well as providing optimized and standardized culturing conditions. Finding an optimal co-culturing system may bring us closer to clinical application of tissue engineered bone grafts.

## Figures and Tables

**Table 1:** Advantages and disadvantages of HUVECs application for pre-vascularization studies in tissue engineering.

Pros	Cons
Non-invasive harvesting method from “medical waste”	Site-specific phenotype
Can be easily isolated in high numbers	Immunogenicity
A large number of published studies = comparable results	Impossibility of autotransplantation in adult patients
